# Association between clinical features and Hsp70 plasma levels in adults with non-segmental vitiligo: a cross-sectional study^⋆^

**DOI:** 10.1016/j.abd.2022.09.002

**Published:** 2023-02-28

**Authors:** Helena Zenedin Marchioro, Caio César Silva de Castro, Isabella Gizzi Jiacomini, Hélio Amante Miot

**Affiliations:** a Department of Dermatology, Hospital Santa Casa de Curitiba, Curitiba, PR, Brazil; a Department of Dermatology, Hospital Santa Casa de Curitiba, Curitiba, PR, Brazil; b Department of Dermatology, Pontifícia Universidade Católica do Paraná, Curitiba, PR, Brazil; c Department of Basic Pathology, Division of Biological Sciences, Universidade Federal do Paraná, Curitiba, PR, Brazil; d Department of Dermatology and Radiotherapy, Faculty of Medicine, Universidade Estadual Paulista, Botucatu, SP, Brazil

Dear Editor,

Vitiligo is a systemic autoimmune disease, characterized by skin depigmentation, which affects 0.57% of the Brazilian population and corresponds to 1.6% of dermatological consultations in Brazil.^1^ Its pathogenesis involves genetic predisposition, the sensitivity of melanocytes to oxidative stress, intercellular adhesion failure of melanocytes, and immune response activation, which justifies the higher frequency of concomitant autoimmune diseases, such as thyroid disease, diabetes mellitus, and systemic lupus erythematosus.^2^

Heat shock protein 70 (Hsp70) is a key molecule in the innate immune response of vitiligo, constituting an intracellular chaperone, responsible for the correct folding of proteins in the endoplasmic reticulum and for preventing the aggregation of misfolded proteins. It exists naturally in tissues and can be induced by cellular stress. In the pathophysiology of vitiligo, after an initial trigger (ultraviolet radiation, trauma, or exposure to phenolic compounds), melanocytes undergo an oxidative stress process, which induces Hsp70 synthesis. After that, Hsp70 is secreted into the extracellular environment leading to the activation of dendritic cells, which are transformed into antigen-presenting cells, which activate T lymphocytes, initiating the adaptive immune response. Therefore, Hsp70 is considered a link between the innate and adaptive immune responses in vitiligo.^3^ Studies in animal models have shown that Hsp70 is not only necessary but sufficient to trigger depigmentation in vitiligo-prone mice.^4^

While tissue levels of Hsp70 are increased in active vitiligo, there are no studies that have investigated the circulating levels of the protein in these patients.^5^

A cross-sectional study was carried out in Curitiba, state of Paraná (Brazil), between November 2020 and February 2022, aiming to determine plasma levels of Hsp70 in adults with non-segmental vitiligo not yet submitted to immunosuppression, without any rheumatological disease, cancer or inflammatory bowel disease (IBD), so as not to confuse Hsp70 levels with inflammatory mediators. Clinical data and peripheral blood samples were collected from the participants during the medical visit. The Hsp70 ELISA kit (ADI-EKS-700B, Enzo Life Sciences, New York, USA) was used to estimate circulating Hsp70 levels. The obtained values were compared between subgroups regarding disease activity: stable (>1 year), progression, and regression (undergoing repigmentation); as well as in relation to clinical extension. Disease activity was determined by patient self-report. Disease extent was estimated using the “Vitiligo Calculator” application (https://www.vitiligo-calculator.com), which uses the VES (Vitiligo Extent Score) instrument validated by van Geel et al. VES is based on standardized images with different degrees of skin involvement, in 19 different topographies. Numerically, VES can vary from zero to 100 and shows a high correlation with the vitiligo area scoring index (VASI).^6^

A total of 89 participants were included in the study, and their main clinical and demographic data are shown in Table 1. Most individuals showed disease progression at their inclusion in the study, with wide variability in clinical extent of disease (VES ranged from 0.04 to 71.06), and plasma levels of Hsp70 (ranging from 1.82 to 27.8 ng/mL). The Hsp70 cutoff value was defined as 2.8 ng/mL for comparison between the subgroups. This value was defined as the best entropic separation between individuals with and without disease activity, since there is no standard of normality in the literature, both in the general population and for vitiligo patients.

**Table 1:** Main clinical and demographic data of adult patients with non-segmental vitiligo (n = 89).

Variables	Values	
**Sex, n (%)**		
Male	30	33.7%
Female	59	66.3%
**Age (years), mean (SD)**	48.8	10.6
**Phototype, n (%)**		
I and II	22	24.7%
III	40	44.9%
IV and V	27	30.3%
Level of schooling, n (%)		
**Elementary school**	23	25.8%
High School	29	32.6%
Higher Education	37	41.6%
**BMI (kg/m**^**2**^**), mean (SD)**	27.1	4.6
**Comorbidities, n (%)**		
Arterial hypertension	18	20.2%
Diabetes	8	9.0%
Thyroid disease	20	22.5%
Depression	17	19.1%
Anxiety	24	27.0%
Dyslipidemia	12	13.5%
**Age at onset of vitiligo, median (IQD)**	38	15.8
**Duration of disease (years), mean (SD)**	10	14
**Current treatments, n (%)**		
Topical corticosteroids	12	13.5%
Topical calcineurin inhibitor	42	47.2%
PUVA	15	16.9%
NB-UVB	17	19.1%
**Disease activity, n (%)**		
Regression	13	14.6%
Stable	14	15.7%
Progression	62	69.7%
**VES, median (IQD)**	1.5	4.3
**Hsp70 (ng/mL), median (IQD)**	2.6	1.4
**Hsp70 > 2.8 ng/mL, n (%)**	38	43%

VES, Vitiligo Extent Score; BMI, Body Mass Index; SD, Standard Deviation; IQD, Interquartile deviation, PUVA, psoralen ultraviolet A, NB-UVB, narrowband utraviolet B.

There was no direct correlation between Hsp70 levels and VES (rho = 0.08; p = 0.461), nor between Hsp70 and disease duration, BMI, age, and phototype (rho < 0.16; p > 0.1).

When considering the normal cutoff value for Hsp70 as 2.8 ng/mL, 76.9% of patients with vitiligo undergoing regression showed altered values, while this occurred in only 35.7% of patients with stable disease and 37.1% of those in progression (p = 0.028). This behavior was confirmed even when adjusted for sex, age, extent of disease, and phototype (Table 2).

**Table 2:** Multivariate logistic model of the association of elevated plasma levels of Hsp70 (>2.8 ng/mL) with clinical and demographic data of adult patients with non-segmental vitiligo (n = 89).

Variable	Frequency	Odds Ratio (95% CI)	p-value
**Sex (male)**	30 (34%)	1.40 (0.51–3.82)	0.513
**Age (> 50 years)**	43 (48%)	1.23 (0.50–3.02)	0.650
**Phototype**			0.370
I and II	22 (25%)	1 (-)	
III	40 (45%)	1.38 (0.42–4.53)	
IV and V	27 (30%)	2.04 (0.60–6.94)	
**VES (>3)**	28 (32%)	1.36 (0.49–3.77)	0.550
**Disease activity**			**0.026**
Regression	13 (15%)	1 (‒)	
Stable	14 (16%)	0.17 (0.03–0.94)	
Progression	62 (39%)	0.16 (0.04–0.69)	

VES, Vitiligo Extent Score; 95% CI, 95% confidence interval.

Altered Hsp70 values were not associated with the assessed comorbidities (p > 0.40). Among current treatments, oral corticosteroids, methotrexate, and PUVA (psoralen and UVA) were not associated with Hsp70 levels (p > 0.20); however, participants undergoing phototherapy with UVB narrow band (NB-UVB) showed a higher frequency of positivity for Hsp70: 71% vs. 36% (OR = 4.2; 95% CI 1.3–13.4; p = 0.010).

Although some studies have shown an increase in tissue Hsp70 levels in lesional and perilesional skin of active vitiligo, plasma levels of this protein in patients with the disease had not been evaluated to date, nor had it been attempted to correlate them with disease activity.^5^ In the present research, no correlation was found between plasma levels of Hsp70 and disease extension; however, Hsp70 positivity was associated with regressive vitiligo.

This finding suggests there is no alignment between the behavior of plasma Hsp70 levels and the tissue expression of Hsp70, limiting its use as a direct marker of disease severity. In fact, other inflammatory dermatoses such as psoriasis and atopic dermatitis do not show a strong correlation between plasma markers and disease extension, either. To date, there are no studies on vitiligo in which plasma Hsp70 levels have been measured and the tissue Hsp70 concentration of the affected skin has been estimated in the same individual.

However, it should be considered that Hsp70 may play different roles according to the stage of the disease. For instance, in rheumatoid arthritis, different studies have shown contradictory roles for Hsp70, that is, both in promoting inflammation, inducing the production of pro-inflammatory cytokines, and in its modulation, stimulating the formation of regulatory T cells (Tregs) and interrupting disease activity.^7^ In inflammatory bowel diseases (IBD), there is evidence that Hsp70 expression is increased in the intestinal mucosa, but most studies point to a protective role, in which the local increase in Hsp70 levels was associated with a reduction in disease activity, in experimental models.^8^, ^9^ Moreover, an important role for Hsp70 in suppressing the immune response through the formation of regulatory B cells (Bregs) has been demonstrated in an experimental study using animal models of autoimmune thyroiditis and IBD.^10^

The pathophysiological model of vitiligo is a complex one. Regulatory T cell (Tregs) dysfunction is one of the maintenance pathways of autoimmunity in vitiligo. However, whether there is any role for Bregs in vitiligo and whether circulating Hsp70 participates in this modulation is yet to be elucidated.

Individuals undergoing treatment with Nb-UVB showed higher Hsp70 positivity. Furthermore, when using the cutoff point of 2.8 ng/mL, plasma levels of Hsp70 were associated with regressing vitiligo. It is possible that Hsp70 plays different roles in vitiligo, depending on the stage of the disease, or that Nb-UVB phototherapy itself induces an increase in circulating Hsp70 levels. The association between UVB exposure, vitamin D levels, phototherapy-induced oxidative stress, and plasma Hsp70 levels should be explored with appropriate designs.

The present study has limitations associated with the lack of confirmation of tissue expression of Hsp70 in the participants. Further investigations exploring plasma Hsp70 levels and the immune tolerance mechanisms at different stages of disease activity are needed to clarify the role of circulating Hsp70 levels in vitiligo.

In conclusion, plasma levels of Hsp70 did not directly correlate with the extent of vitiligo; however, greater positivity was identified in participants with the regressive forms of the disease.

## Approval by the Ethics Committee

This project was approved by the Ethics Committee of Pontifícia Universidade Católica do Paraná (Counsel number 4,333,389).

## Financial support

FUNADERM.

## Authors' contributions

Helena Zenedin Marchioro: Design of the study; data collection; analysis of the results; manuscript writing; review and approval of the final text.

Caio César Silva de Castro: Design of the study; analysis of the results; manuscript writing; review and approval of the final text.

Isabella Gizzi Jiacomini: Data collection, analysis of the results; review and final approval of the text.

Hélio Amante Miot: Design and planning of the study; collection, analysis and interpretation of data; statistical analysis; manuscript writing; critical review of the literature; critical review of the manuscript; approval of the final version of the manuscript.

## Conflicts of interest

Helena Zenedin Marchioro: None declared.

Caio César Silva de Castro: Ache – speaker, Advisory Board; Sun pharma – Advisory Board; Abbvie – Advisory Board; Pfizer – clinical trial.

Isabella Gizzi Jiacomini: None declared.

Hélio Amante Miot: None declared.
